# Estrogen regulates luminal progenitor cell differentiation through *H19* gene expression

**DOI:** 10.1530/ERC-15-0105

**Published:** 2015-08

**Authors:** Pratima Basak, Sumanta Chatterjee, Steven Weger, M Christine Bruce, Leigh C Murphy, Afshin Raouf

**Affiliations:** 1 Department of Immunology, University of Manitoba, 471 Apotex Centre 750 McDermot Avenue, Winnipeg, Manitoba, R3E 0T5, Canada; 2 Manitoba Institute of Cell Biology, 675 McDermot Avenue, Winnipeg, Manitoba, R3E 0V9, Canada; 3 Department of Biochemistry and Medical Genetics, University of Manitoba, Winnipeg, Manitoba, R3E 0W2, Canada

**Keywords:** ERα, *H19*, luminal progenitors, ER+ breast cancer cells

## Abstract

Although the role of estrogen signaling in breast cancer development has been extensively studied, the mechanisms that regulate the indispensable role of estrogen in normal mammary gland development have not been well studied. Because of the unavailability of culture system to maintain estrogen-receptor-positive (ERα^+^) cells *in vitro*, the molecular mechanisms that regulate estrogen/ERα signaling in the normal human breast are unknown. In the present study, we examined the effects of estrogen signaling on ERα^+^ human luminal progenitors using a modified matrigel assay and found that estrogen signaling increased the expansion potential of these progenitors. Furthermore, we found that blocking ERα attenuated luminal progenitor expansion and decreased the luminal colony-forming potential of these progenitors. Additionally, blocking ERα decreased *H19* expression in the luminal progenitors and led to the development of smaller luminal colonies. We further showed that knocking down the *H19* gene in the luminal progenitors significantly decreased the colony-forming potential of the luminal progenitors, and this phenotype could not be rescued by the addition of estrogen. Lastly, we explored the clinical relevance of the estrogen–*H19* signaling axis in breast tumors and found that ERα^+^ tumors exhibited a higher expression of *H19* as compared with ERα^−^ tumors and that *H19* expression showed a positive correlation with ERα expression in those tumors. Taken together, the present results indicate that the estrogen–ERα–*H19* signaling axis plays a role in regulating the proliferation and differentiation potentials of the normal luminal progenitors and that this signaling network may also be important in the development of ER^+^ breast cancer tumors.

## Introduction

Estrogen signaling through estrogen receptor α (ERα) is pivotal to the survival and maintenance of ERα^+^ breast cancer tumors. Furthermore, the estrogen–ERα signaling axis has been shown to be indispensable to mammary gland development, seeing as ERα knockout or ovariectomized mice only develop rudimentary breast structures (Daniel *et al*. 1987, Bocchinfuso & Korach 1997*a*). The results of previous investigations have indicated that the nature of the estrogen signaling network changes dramatically during human breast tumorigenesis. This is evidenced by the fact that increased ERα expression in the normal breast increases the risk of developing breast cancer and the fact that elevated ERα expression is an early event that occurs in premalignant lesions (Clarke *et al*. 1997, Clemons & Goss 2001, Lee *et al*. 2006). These observations indicate that alterations to estrogen signaling and its molecular targets may serve as early, premalignant changes in breast epithelial cells. However, the exact mechanisms by which estrogen signaling regulates the proliferation and differentiation of normal human breast cells are unknown.

Current results indicate that estrogen acts as an indirect mitogen in normal breast epithelial cells (Lippman *et al*. 1977, Clarke *et al*. 1997, Shoker *et al*. 1999). This concept is based on the observation that although increased ERα expression and an increased number of ERα^+^ cells can be observed during the menstrual cycle in humans (Soderqvist *et al*. 1997), few, if any, ERα^+^ cells also show staining for proliferation markers (Clarke *et al*. 1997, Anderson *et al*. 1998). Also, the progesterone receptor (PR, a classical ERα target in breast cancer cells) and ERα mostly show divergent expression profiles in the normal breast (Hilton *et al*. 2012). On the basis of these observations, it can be postulated that ERα^+^ cells act in a paracrine manner to stimulate the proliferation of ERα^−^ cells (Mallepell *et al*. 2006). However, no functional studies have been conducted to examine the direct effects of estrogen signaling on normal ERα^+^ human breast cells. Therefore, our understanding of the molecular mechanisms that are regulated by estrogen signaling in the breast is primarily based on ERα^+^ human breast cancer cell lines or xenografts in athymic mice. Because of the lack of ERα^+^ non-malignant cell lines (Anderson *et al*. 1998), a functional analysis of primary ERα^+^ cells obtained from human breast tissue is required in order to study the mechanisms by which the estrogen–ERα signaling network regulates mammary gland development.

Human breast tissue is made up of luminal and myoepithelial cells that are continuously produced from bipotent and lineage-restricted progenitors, which are themselves generated from a rare population of breast stem cells (Eirew *et al*. 2008, 2010, Raouf *et al*. 2008). The transcriptome profiling of these different progenitor subtypes has revealed that the undifferentiated bipotent progenitors showed high enrichment for PR transcripts, whereas the luminal progenitors showed enrichment for the ERα transcripts (Raouf *et al*. 2008). The results of recent studies in human and mouse mammary glands have provided some insights into the potential role of progesterone in the regulation of bipotent progenitors and stem cells (Joshi *et al*. 2010, Hilton *et al*. 2014). More recently, it has been demonstrated that ERα^+^ human breast cells are able to grow and generate colonies *in vitro* and mammary structures *in vivo* that are restricted to the luminal lineage (Honeth *et al*. 2014). However, there is a gap in our knowledge about the molecular mechanisms by which estrogen signaling regulates the proliferation and differentiation potentials of these ERα^+^ luminal progenitors. Such knowledge would be important for understanding how known breast cancer oncogenes function in healthy ERα^+^ cells and how alterations to their functions can lead to breast tumor development. In this context, *H19* is an estrogen-regulated gene (Adriaenssens *et al*. 1999) that was previously identified as a breast tumor oncogene (Berteaux *et al*. 2005). *H19* is a long non-coding RNA (lncRNA) that harbors the microRNA-675 in its first exon and has been shown to regulate estrogen-induced proliferation of ERα^+^ breast cancer cells (Lottin *et al*. 2002, Cai & Cullen 2007, Keniry *et al*. 2012). However, it is not clear if the *H19* gene plays a similar role in regulating the proliferation of healthy ERα^+^ cells.

In the present study, we therefore used a modified *in vitro* matrigel assay to culture primary human ERα^+^ luminal progenitors under estrogen-depleted growth conditions in order to directly examine the effect of estrogen signaling on these cells at the molecular and cellular levels. Using this assay and the colony-forming cell (CFC) assay, we found that estrogen–ERα signaling enhances the proliferation and differentiation potentials of the luminal progenitors through increased *H19* gene expression. Furthermore, we provide evidence that *H19* is highly expressed in ERα^+^ breast cancer tumors and that its expression positively correlates with ERα expression in these tumors. These findings are significant because increased estrogen signaling has been demonstrated to be a risk factor for developing ERα^+^ breast tumors; therefore, understanding the molecular mechanisms that govern ERα and estrogen signaling in healthy breast cells provides insights into how alterations to this signaling axis may lead to the development of premalignant lesions.

## Materials and methods

For more detailed descriptions, see the Supplementary Materials and methods (see section on supplementary data given at the end of this article).

### Isolation of luminal and bipotent progenitors

Luminal and bipotent progenitors were isolated from reduction mammoplasty samples that were obtained through informed, written patient consent in accordance with the University of Manitoba's Health Research Ethics Board (REB no. H2010:292) policies. Breast reduction samples were enzymatically dissociated, and the organoid-enriched fractions were obtained as described previously (Raouf & Sun 2013). Single-cell suspensions were prepared from these organoid-enriched fractions and were placed in co-cultures with irradiated 3T3 mouse fibroblasts (ATCC, X-irradiated at 30 Grey, i3T3) in SF7 media (Stingl *et al*. 1998, Raouf *et al*. 2008, Raouf & Sun 2013) supplemented with 5% fetal bovine serum (FBS). After 3 days, adherent cells were made into single-cell suspensions as described previously (Raouf & Sun 2013) and were blocked with 10% human serum. Cells were then stained with anti-epithelial cell adhesion molecule (EpCAM, 1:100 dilution; StemCell Technologies, Vancouver, BC, Canada) conjugated to phycoerythrin (PE) and anti-α6 integrin (CD49f) conjugated to Alexa Fluor 647 (1:100 dilution; Biolegend, San Diego, CA, USA). For isotype controls, IgG antibodies directly conjugated to PE or allophycocyanin (APC) were used (both from BD Biosciences, San Jose, CA, USA). Propidium iodide (PI, 1 mg/ml) was added to each sample to distinguish live cells. PI^−^EpCAM^high^CD49f^low^ (luminal progenitors) or PI^−^EpCAM^low^CD49^bright^ (bipotent progenitors) cells were sorted via fluorescence-activated cell sorting (FACS).

### Matrigel cultures

For gene expression studies, the luminal or bipotent progenitors (2.0–3.5×10^4^ cells/well) were combined with i3T3 cells (2.0×10^5^ cells/well) and placed in 96-well plates containing 50 μl of polymerized, growth-factor-reduced, and phenol-red-free (PRF) matrigel (BD Biosciences). Cells were grown in complete media (PRF-SF7 supplemented with bovine pituitary extract (BPE) at 100 μg/ml) for 7 days and then cultured in estrogen-depleted growth media (PRF-DMEM supplemented with 5% charcoal-stripped serum (v/v) (Weitsman *et al*. 2006)) for 48 h. Thereafter, the cultures were treated with 17β-estradiol (E_2_, 10 nM) or ethanol (EtOH) for an additional 24 h, at which point the gels were dissociated and RNA was extracted from the cells using TRIzol Reagent (Life Technologies). For some of the experiments, two individual reduction mammoplasty samples were pooled to yield sufficient numbers of luminal progenitors for carrying out the experiments.

To quantify the effects of E_2_ on luminal progenitor expansion and differentiation, isolated progenitors were placed in matrigel cultures with 5.0×10^4^ cells/well or in co-cultures with i3T3 (2.0–3.5×10^4^ luminal progenitors combined with 2.0×10^5^ i3T3 cells per well in a 96-well plate). After 7 days, the growth media was replaced with PRF-SF7 supplemented with E_2_ (10 nM) or E_2_ (10 nM) plus ICI (500 nM) or with ethanol. After an additional 7 days, gels were dissociated and cells were placed in CFC assays as described below. The frequencies of luminal progenitors before and after matrigel cultures were ascertained by CFC assays as described in the next section.

### CFC assays

Luminal and bipotent progenitors were examined using CFC assays essentially as described previously (Raouf *et al*. 2008) with the following modifications: 1000 progenitors were combined with 8.0×10^4^ i3T3 cells and were cultured in collagen-treated six-well tissue culture plates using SF7 growth medium supplemented with 2% FBS for 7–10 days. Colonies were fixed with an acetone:methanol (1:1 ratio) solution and stained with a crystal violet solution (0.05% w/v). Some CFC assays were set up as described above, except that PRF SF7 medium supplemented with 2% FBS was used. After 18 h, growth media were replaced with PRF SF7 media supplemented with E_2_ (10 nM) or E_2_ plus ICI 182,780 (ICI, at the indicated concentrations), EtOH, or ICI only (at the indicated concentrations) for 6 days. Subsequently, the colonies were fixed and stained as described above. The colony numbers were obtained using an inverted microscope (Evos XL Core), and colony size (number of cells/colony) was determined (analyzed using Adobe Acrobat X Pro, San Jose, CA, USA).

### *H19*-deficient luminal progenitors

Single-cell suspensions prepared from the organoid-enriched fractions (1×10^6^ cells) were placed in co-cultures with 1.6×10^5^ i3T3 cells in SF7 media supplemented with 5% FBS. After 24 h, cells were infected separately with 1×10^7^ lentiviral particles prepared from a pool of three different pGIPZ-puro-GFP plasmids containing short hairpin (sh)-RNA that target the *H19* RNA or a pGIPZ-puro-GFP vector that expresses a scrambled sh-RNA fragment (Thermo Fisher, Waltham, MA, USA; for details on lentivirus production and infection, see Raouf *et al*. (2008)). After 4 days, the GFP+ luminal progenitors were isolated via FACS and placed in CFC assays. For some of the experiments, the growth media were supplemented with either 10 nM E_2_, E_2_ plus ICI (500 nM), or EtOH after 18 h, cultures were grown for 6 additional days, and colonies were fixed and analyzed as described above.

### Overexpression or knockdown of ERα and ERβ in breast cancer cells

ERα was expressed in the MDA-MB231 by transfecting the pCDNA 3.1-ERα vector into the cells using the conventional calcium chloride method (Wigler *et al*. 1977, Chen & Okayama 1987). The transfected cells were selected using geneticin (2.5 μg/ml, Sigma–Aldrich) and then grown in estrogen-depleted growth media for 48 h, at which point cells were treated with E_2_ (10 nM) or ethanol-supplemented growth media for 24 h. ERα expression in the MCF7 cells was knocked down by infection with lentivirus prepared from pGIPZ-sh-ERα or pGIPZ-scrambled control (Thermo Fisher) for 4 h. Three separate pGIPZ-sh-ERα constructs (1, 2, and 3) were used. After 48 h, the transduced cells were puromycin selected (2 μg/ml, Sigma–Aldrich) for 72 h. The MDA-MB231-ERβ cells (Murphy *et al*. 2005) were placed in estrogen-depleted growth media for 48 h and then grown in PRF-DMEM supplemented with 5% CS-FBS, doxycycline (2 μg/ml; Sigma), and E_2_ (10 nM) or EtOH for 24 h.

### Chromatin immunoprecipitation assay

MCF7 cells were grown in estrogen-depleted growth media for 48 h and treated with either E_2_ (10 nM) at 1 h, 3 h, 6 h, and 24 h or with ethanol for 24 h. ERα chromatin immunoprecipitation (ChIP) was carried out as described previously (Schmidt *et al*. 2009, Ross-Innes *et al*. 2012). Equal amounts of ChIP DNA were analyzed by qPCR for the estrogen responsive elements (EREs) in the *H19* promoter using the oligonucleotides indicated in Supplementary Table 1, see section on supplementary data given at the end of this article. Melting temperatures were examined to ensure that a singular amplicon was obtained from each primer set. The binding enrichment to the *H19* promoter regions was obtained using the percentage of ChIP DNA (relative to input DNA) normalized to a nonspecific control region of the genome, as described previously (Holmes *et al*. 2008).

### Selection of archival human breast tumors samples

All of the human breast cancers tumors were obtained from the Manitoba Breast Tumour Bank (MBTB) (Watson *et al*. 1996). All of the samples were obtained and used in accordance with the University of Manitoba's Health Research Ethics Board (REB no. H2001:083). ERα and PR protein levels in each tumor were determined using the ligand-binding assay, where >3 fmol/mg of protein was considered positive (Watson *et al*. 1996, Barnes *et al*. 2008). A total of 70 tumor samples were selected, of which 39 were deemed to be ER^+^ and 31 were categorized as ER^−^ (i.e., <3 fmol/mg protein). RNA was extracted from frozen tumor sections using TRIzol Reagent, treated with DNase, and turned into cDNA.

### Statistical analysis

The differential expression of genes was determined using the two-tailed Student's *t*-test, and the differential expression of *H19* in the breast tumor samples was determined using the two-sided Mann–Whitney rank test. The statistical significance of an increased dose of ICI on *H19* transcript levels was assessed by ANOVA. All of the tests were conducted with the GraphPad Prism 4.02 program (GraphPad, La Jolla, CA, USA). The correlation between the ERα transcript levels and *H19* expression was determined using Fisher's exact test. For this purpose, the best-fit line (Microsoft Excel) was forced through the origin, because more than half of the data points fell close to the origin of the scatter plot.

## Results

### EpCAM^+^CD49f^low^ subset of human breast epithelial cells are enriched for ERα^+^ luminal progenitors

**Figure 1:**
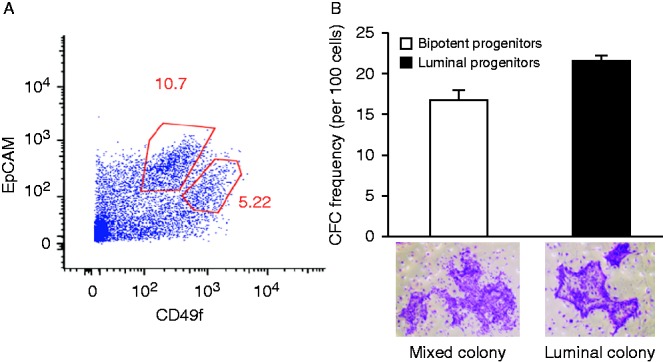
ERα is strongly expressed in luminal progenitors. (A) Luminal (EpCAM^brig^ ^ht^ CD49f^low^) and bipotent (EpCAM^low^CD49f^bright^) progenitors were isolated from reduction mammoplasty samples using FACS. (B) CFC assays were used to determine the purity of different progenitor subtypes. As shown, 21.6% of the EpCAM^bright^CD49f^low^ cells formed pure luminal colonies, whereas 16.7% of the EpCAM^low^ CD49f^bright^ cells formed mixed colonies (*n*=3). Representative luminal and mixed colonies are shown in the photographs.

Luminal and bipotent progenitors were isolated from reduction mammoplasty samples by FACS (Fig. 1A). With this strategy, EpCAM^+^CD49f^low^ cells were enriched for the luminal progenitors (21.6% purity), whereas EpCAM^low^CD49f^+^ cells were enriched for the bipotent progenitors (16.7% purity) (Fig. 1A and B). The frequency of the luminal colonies was determined by CFC assay, an *in vitro* clonal assay that provides a prospective measure of luminal progenitor numbers in the initial sample and their differentiation potential to form mature luminal colonies (i.e., through the quantification of colony size). Next, we confirmed the high expression of ERα in the freshly isolated luminal progenitors as compared with the bipotent progenitors through immunofluorescence staining (Supplementary Figure 1A, see section on supplementary data given at the end of this article). The luminal phenotype of the cells in the colonies was determined by measuring cytokeratin 18 expression (Supplementary Figure 1B).

### Estrogen–ERα signaling enhances luminal progenitor expansion and differentiation

**Figure 2:**
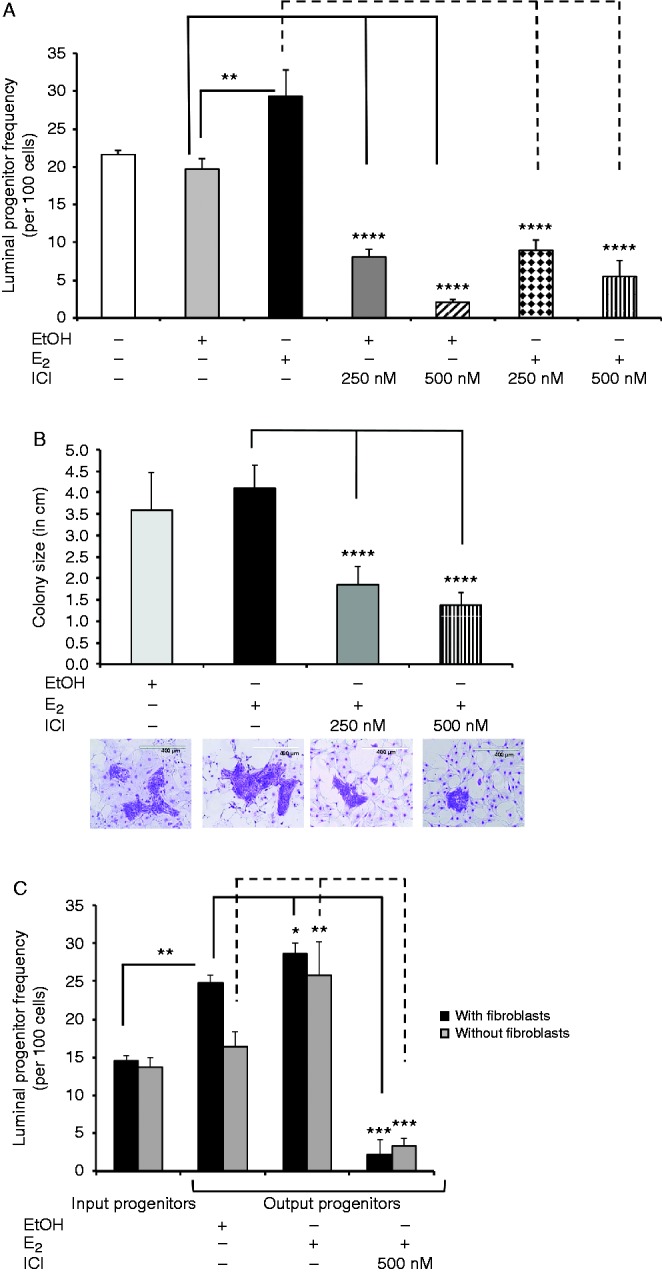
Estrogen signaling enhances luminal progenitor proliferation. (A) Luminal progenitors were examined using CFC assays and were either treated with E_2_, EtOH, E_2_ plus ICI, or EtOH plus ICI at the indicated concentrations. The CFC cultures were fixed and stained, and the colony numbers were quantified (*n*=3). As shown, in the presence of E_2_, the colony-forming capacity of luminal progenitors was increased (1.48-fold as compared to EtOH), and blocking ERα significantly attenuated this induction. (B) Luminal progenitors were treated as in (A), and colony size (i.e., the ability of the luminal progenitors to form mature luminal cells) was determined by counting the number of cells in each colony. As shown, ICI treatment significantly reduced colony size in a dose-dependent manner. Images show representative colonies from each treatment. (C) Luminal progenitors were placed in matrigel cultures with or without irradiated fibroblasts (i3T3) in complete medium for 7 days and were then treated with E_2_, E_2_ plus ICI, or EtOH for an additional 7 days. Thereafter, the frequency of progenitors was assessed via CFC assay. In matrigels without i3T3, E_2_ significantly increased luminal progenitor frequency, which was circumvented with ICI. Interestingly, the addition of i3T3 alone increased progenitor cell numbers, and the addition of E_2_ had a small but statistically significant effect. Aliquots from the same samples were placed in the CFC assays before the matrigel cultures to give the starting luminal progenitor frequency. *P<0.05, **P<0.005, ***P<0.0001, ****P<0.00001.

Although estrogen signaling is indispensable for mammary gland development, the precise mechanisms that underlie its action are not clear (Bocchinfuso & Korach 1997*b*, Bocchinfuso *et al*. 2000). To address this issue, we examined whether estrogen signaling plays a role in regulating the proliferation and differentiation of ERα^+^ human luminal progenitors. For this purpose, CFC assays were set up either in the presence of E_2_ or increasing concentrations of fulvestrant (ICI 182,780), a specific estrogen receptor antagonist (Fig. 2A and Supplementary Figure 2A, see section on supplementary data given at the end of this article). The addition of E_2_ caused a modest yet statistically significant (1.48-fold) increase in the luminal colony numbers. However, the addition of 250 nM or 500 nM ICI in the presence of E_2_ decreased colony-forming potential of the luminal progenitors by 3.2- and 5.3-fold respectively (Fig. 2A). Because CFC assays were unamenable to estrogen-deprived culture conditions, these experiments were performed in complete media, which would contain sources of estrogens. It is therefore not surprising that only a modest increase in the number of colonies was observed in the presence of additional E_2_. Interestingly, we also observed that increased concentrations of ICI led to significantly decreased luminal numbers (Supplementary Figure 2A) and that no colonies were observed in the presence of higher concentrations of ICI. Colony size is a measure of the potential of luminal progenitors to generate mature luminal colonies (Stingl *et al*. 1998). We therefore quantified the number of cells per colony as a prospective measure of the differentiation potential of luminal progenitors. Interestingly, in the presence of ICI, we observed a significant reduction in luminal colony size (Fig. 2B). It is noteworthy that the addition of E_2_ did not increase colony size. We posit that this was because of the existing levels of estrogens in the growth media, and the addition of E_2_ to these cultures was therefore ineffective in enhancing the luminal colony size. These results indicate that estrogen–ERα signaling is essential to the proliferation and differentiation of luminal progenitors.

The examination of estrogen signaling at the molecular level in luminal progenitors requires the luminal progenitors to be placed into estrogen-depleted growthconditions. This was accomplished by placing isolated luminal progenitors into matrigel cultures along with irradiated mouse embryonic fibroblasts (i3T3) for 7 days, after which the cultures were treated with E_2_. Using immunofluorescence staining and FACS, we determined that cells in the E_2_-treated gels showed a 3.2-fold increased expression of ERα (Supplementary Figure 1C and D). Next, we examined the frequency of luminal progenitors in the matrigel cultures that had been treated with E_2_ or E_2_ plus ICI. Using CFC assays, we found that compared with the EtOH-treated controls, E_2_ stimulation increased the frequency of luminal progenitors by 13%, which was completely attenuated in the presence of ICI (Fig. 2C, black bars, input luminal progenitors as compared with output progenitors) and exhibited a twofold increase as compared with the input controls. Also, we observed that placing the luminal progenitors in matrigel cultures with i3T3 cells plus EtOH significantly increased the frequency of the progenitors (Fig. 2C, black bars, input as compared with the ethanol controls), which indicates that either fibroblasts or ethanol alone could increase the frequency of luminal progenitors and that estrogen may not act directly on luminal progenitors. It is noteworthy that in the presence of ICI, the colony-forming potential of the luminal progenitors was less than that of the cultures treated with ethanol as controls. This observation was most probably a result of the residual estradiol in the matrigel preparation that was present in the ethanol-treated cultures but was completely neutralized in presence of ICI. To examine whether the estrogen-induced expansion of the numbers of luminal progenitors in the matrigel cultures required fibroblasts, matrigel cultures initiated with luminal progenitors without fibroblasts were treated with EtOH, E_2_, or E_2_ plus ICI (Fig. 2C). Luminal progenitor frequency remained the same in the absence of i3T3 plus ethanol, which indicates that fibroblasts alone can increase the frequency of luminal progenitors. However, upon stimulation with E_2_ and in the absence of fibroblasts, luminal progenitor frequency increased by twofold, but this increase was abrogated by ICI. We examined 7-day matrigel cultures that had been initiated with luminal progenitors and determined that as many as 60% of the cells in these gels expressed ERα^+^ (Supplementary Figure 2B and C). We also found that the matrigel treated with E_2_ contained significantly more Ki67^+^ cells as compared with the EtOH-treated gels (Supplementary Figure 2D). To ensure that placing luminal progenitors in matrigel cultures does not alter their phenotype, the expression of CD49f, EpCAM, cytokeratin 18, and cytokeratin 14 was examined in cells obtained from these gels after 7 days of treatment with E_2_ or EtOH. We found no appreciable difference in the expression of these markers in cells grown in EtOH- and E_2_-treated gels (Supplementary Figure 2E). These observations indicate that placing primary human luminal progenitors in matrigel cultures for 14 days does not alter their phenotype.

The results of these experiments indicate that estrogen signaling could increase the proliferation of luminal progenitors and that fibroblasts play a major role in this signaling network. It is interesting that ICI was able to diminish the colony-forming ability of the luminal progenitors in the absence of fibroblasts, which indicates that E_2_ could directly act on ER^+^ luminal progenitors to regulate their proliferation.

### *H19* but not *PR* expression is regulated by estrogen signaling in luminal progenitors

**Figure 3:**
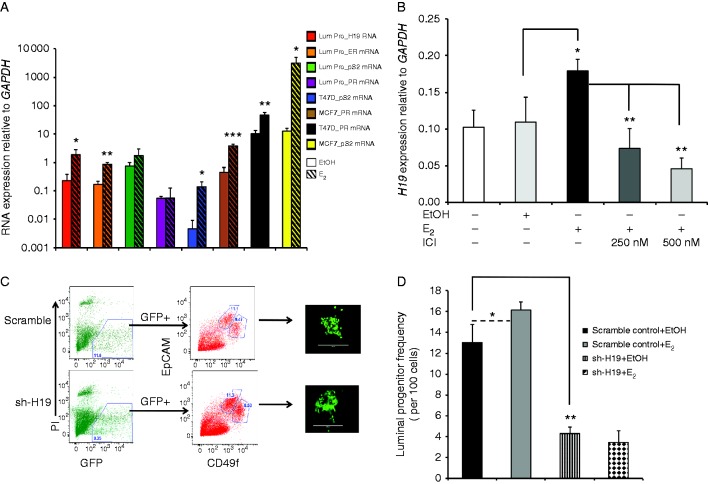
Estrogen signaling enhances luminal progenitor proliferation through *H19*. (A) Luminal progenitors were placed in matrigel assays for 7 days and were then grown in estrogen-depleted growth media for 48 h and were subsequently treated with E_2_ (10 nM) or EtOH for an additional 24 h. qPCR was used to examine the expression of the estrogen target genes (*PR*, *pS2*, ERα, and *H19*) in cells obtained from the gels. Interestingly, no changes in the transcript levels of PR or *pS2* could be detected. However, ERα and *H19* expression levels were increased 5.3- and 8.5-fold respectively in E_2_-exposed luminal progenitors. For comparison, ER^+^ breast cancer cells (MCF7 and T47D) were exposed to EtOH or E_2_ for 24 h, and the expression of estrogen target genes was assessed using qPCR. Solid bars represent transcript expression in EtOH-treated cells, and crossed bars represent E_2_-treated cells. All of the transcript expression levels were normalized to the *GAPDH* levels, and the means and s.d.s are shown (*n*=3). (B) Luminal progenitors were isolated and placed in CFC assays; they were then treated with E_2_ (10 nM), E_2_ plus ICI, or EtOH for 7 days. *H19* expression was examined via qPCR. As shown, in E_2_-stimulated colonies, *H19* expression was increased 1.6-fold, whereas the addition of ICI significantly decreased *H19* expression. (C) Lenti sh-*H19* or lenti sh-scrambled-infected luminal progenitors (GFP+) were isolated via FACS and placed in CFC assays. Scale bars represent 400 μm. Representative pictures show the GFP+ colonies that developed from the transduced luminal progenitors. (D) Average colony counts were used as a prospective measure of the transduced luminal progenitor frequency (*n*=3). Compared with the scrambled control, the sh-*H19*-infected progenitors formed threefold fewer colonies, and the addition of E_2_ had no effect on these transduced progenitors. *P<0.05, **P<0.005, ***P<0.0005.

A number of estrogen target genes, such as *PR*, *pS2*, and *H19*, have been shown to be involved in regulating the proliferation of ER^+^ breast cancer cells (Brown *et al*. 1984, Adriaenssens *et al*. 1999, Cunliffe *et al*. 2003, Carroll *et al*. 2006). However, the nature of the estrogen regulation of these genes in normal ERα^+^ human breast cells has not been explored. Using matrigel cultures, we observed that the transcript expression of *PR* and *pS2* genes showed no significant change in E_2_-stimulated luminal progenitor cultures as compared with the controls, whereas the transcript expression of ERα and *H19* was significantly increased (5.3- and 8.5-fold respectively, Fig. 3A). No significant change in the expression of *H19* or ERα resulting from estrogen treatment could be detected in the bipotent progenitor-initiated matrigel cultures (Supplementary Figure 3A and B, see section on supplementary data given at the end of this article). To the best of our knowledge, this was the first direct measurement of estrogen signaling on isolated normal, non-transformed human ERα^+^ breast cells at the molecular level. Moreover, the addition of E_2_ to the CFC cultures increased *H19* transcript levels significantly, whereas ICI blocked this increase, which indicates that *H19* might be involved in the estrogen–ERα induced expansion of luminal progenitors (Fig. 3B).

### The colony-forming potential of the luminal progenitors requires *H19*

The *H19* gene encodes a lncRNA whose expression in adults is restricted to only a few tissues (e.g., mammary glands) (Pachnis *et al*. 1984, Poirier *et al*. 1991, Doucrasy *et al*. 1993, Dugimont *et al*. 1995, Ariel *et al*. 1997). The biological role of *H19* has been mostly described in relation to *IGF2* signaling; however, its function in breast epithelial cells remains puzzling. Interestingly, *H19* is highly expressed in breast cancer cells (Qiu *et al*. 2013) and may play a role as an oncogene (Lottin *et al*. 2002, Berteaux *et al*. 2005). In addition to autocrine factors (hepatocyte growth factor or fibroblast growth factor 2 (Adriaenssens *et al*. 2002)), *H19* expression is also regulated by estrogen signaling in ERα^+^ breast cancer cells (Adriaenssens *et al*. 1999). We therefore proposed the hypothesis that *H19* might be involved in regulating the estrogen-induced expansion of luminal progenitors. To test this hypothesis, we infected single-cell suspensions prepared from reduction mammoplasty samples with lentivirus that expressed a shRNA against the *H19* RNA (sh-*H19*) or a scrambled control RNA sequence. The transduced luminal progenitors were isolated via FACS (Fig. 3C) and placed in CFC assays in the presence E_2_ or ethanol as a vehicle control. The level of *H19* knockdown in the luminal colonies was assessed using qPCR (Supplementary Figure 4, see section on supplementary data given at the end of this article). Interestingly, the sh-*H19*-transduced human breast cells contained significantly reduced luminal progenitors, which were not rescued with the addition of E_2_ (Fig. 3D). Notably, the loss of *H19* expression led to a threefold decrease in the frequency of luminal progenitors (Fig. 3D). In contrast, the ICI treatment of the luminal progenitors led to a 5.3-fold decrease in the colony-forming ability of the luminal progenitors (Fig. 2A), which indicates that *H19* is an important mediator of estrogen signaling in the luminal progenitors but that other genes may be involved as well. It is interesting that the colonies that developed from the sh-*H19*-infected luminal progenitors contained the same number of cells per colony as the scrambled control-infected progenitors (data not shown). This indicates that *H19* does not play an essential role in the differentiation potential of luminal progenitors.

### Estrogen-induced expression of *H19* is primarily regulated through ERα

As has been shown previously (Adriaenssens *et al*. 1999), we also found that estrogen signaling increases the expression of *H19* in MCF7 and T47D cells (Supplementary Figure 5A, see section on supplementary data given at the end of this article). To ascertain the kinetics of regulation of *H19* expression by estrogen, MCF7 cells were cultured in estrogen-depleted media, and *H19* expression was determined using RT-PCR at various times post-E_2_ stimulation (Supplementary Figure 5B). Interestingly, we found that E_2_ treatment increased *H19* expression as early as 6 h after stimulation and reached its apex within 24 h; this increase was followed by a marked decrease after 48 h. This results indicate that estrogen-induced *H19* expression shows a dynamic pattern of activity that is typical of ERα-regulated gene promoters. Furthermore, increasing doses of ICI led to a sequential decrease in *H19* expression in the MCF7 and T47D cells, which indicates that ERα is directly involved in the regulation of *H19* gene expression (Supplementary Figure 5C and D).

**Figure 4:**
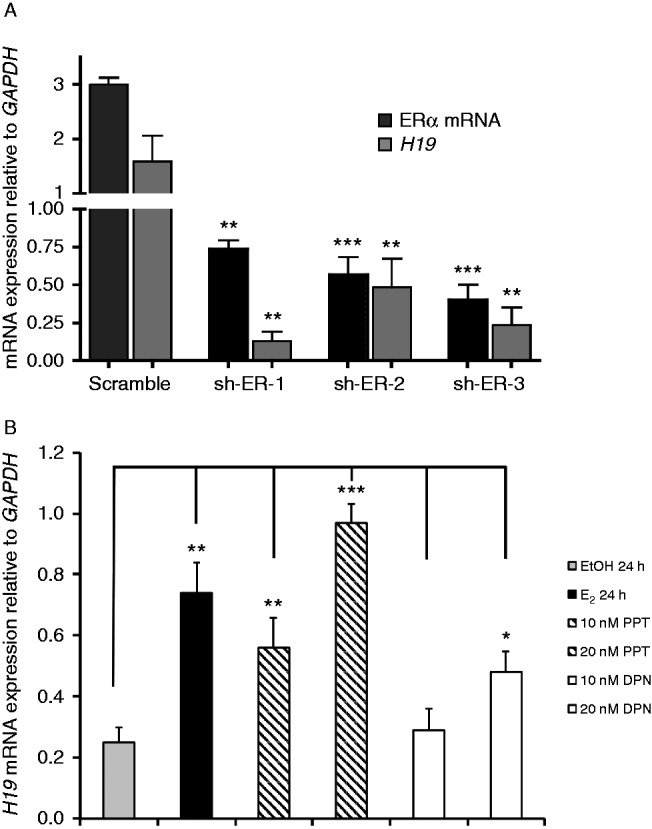
Estrogen-regulated expression of *H19* is mainly regulated through ERα. (A) ERα transcript levels were decreased in MCF7 cells with three different sh-RNA fragments (sh-ER-1, -2, and -3) using lentiviral transduction. ERα and *H19* transcript levels were examined in the transduced cells by qPCR. All of the data are normalized to the *GAPDH* transcript levels. (B) MCF7 cells were grown under estrogen-deprived growth conditions and treated with either EtOH, E_2_, PPT (a selective ERα agonist), or DPN (a selective ERβ agonist) for 24 h. *H19* expression was ascertained using qPCR and was normalized with respect to *GAPDH* expression (*n*=3). Compared with EtOH, as little as 10 nm PPT increased *H19* transcript levels, whereas DPN at the 10 nM concentration level had no effect. However, at 20 nM concentration, both PPT and DPN increased *H19* expression (3.9- and 1.9-fold respectively). *P<0.05, **P<0.005, ***P<0.0005.

Estrogen signaling can be mediated through ERα and ERβ receptors (Murphy & Watson 2002, Clarke *et al*. 2004). To examine the ERα requirement for the estrogen regulation of *H19* expression, lenti shRNA was used to knock down the expression of ERα in MCF7 cells. Compared with the scramble-control-infected cells, loss of ERα expression led to a marked decrease in the expression of *H19* (Fig. 4A). Also, the expression and activation of ERα in MDA-MB231, an ERα^−^ breast cancer cell line, increased *H19* expression by 2.9-fold (Supplementary Figure 6A and B, see section on supplementary data given at the end of this article).

To investigate if ERβ is also involved in estrogen-induced *H19* expression, we used tet-on MDA-MB-231-ERβ cells and found that E_2_ and doxycycline treatment did not increase *H19* expression in these cells (Supplementary Figure 6C and D). Furthermore, we observed that a specific agonist for ERα, PPT (Kraichely *et al*. 2000), was a more potent inducer of *H19* expression (3.9-fold) as compared with an ERβ agonist, DPN (Meyers *et al*. 2001), which provides further support the hypothesis that ERα plays a more dominant role in regulating *H19* expression in MCF7 (Fig. 4B) and in T47D (Supplementary Figure 6E).

### ERα binds to estrogen-responsive elements in the *H19* promoter

We first used inhibitors of transcription and protein synthesis to validate that estrogen induction of *H19* gene expression requires *de novo* transcription without the need for *de novo* protein synthesis (Supplementary Figure 7A, B, C, D and E, see section on supplementary data given at the end of this article).

**Figure 5:**
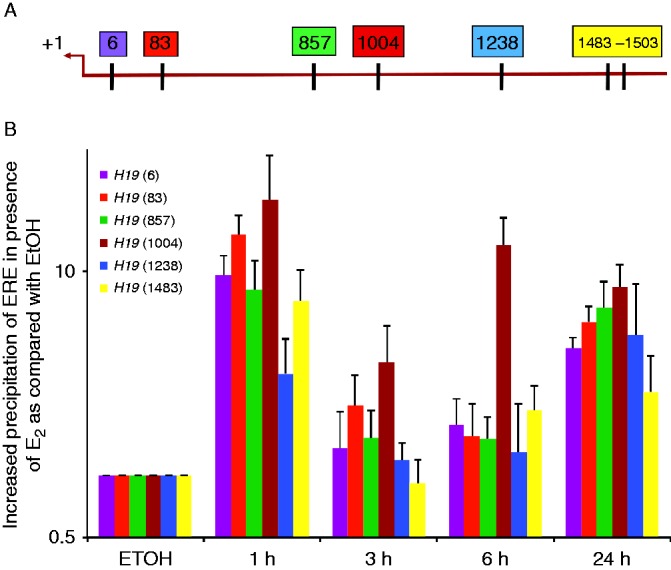
ERα binds to *H19* promoter through EREs. (A) This figure shows the location of EREs within 1500 base pairs of the transcription start site (TSS, +1) of the *H19* proximal promoter. Each box is representative of one ERE half-site, and the number within each box shows its location away from the TSS. (B) Chromatin immunoprecipitation was performed to examine the ERα occupancy of each ERE. MCF7 cells were grown in estrogen-depleted media and treated either with EtOH or E_2_ for the indicated times. ERα bound to each ERE was quantified using qPCR. As shown, ERα binding to the *H19* promoter was detectable after 1 h of exposure to E_2_.

The analysis of the *H19* proximal promoter and enhancer region revealed seven ERα response elements half-sites (ERE, AGGTCA) (Carroll *et al*. 2006) within 1500 base pairs (bp) of the transcription start site (TSS) (Fig. 5A). To ascertain if ERα is able to bind to any or all of these seven ERE half-sites, we performed ChIP assays. For this purpose, DNA-bound ERα fragments were precipitated after 3, 6, and 24 h of exposure to estradiol or ethanol as a vehicle control. Because of the close proximity of the EREs located at 1483–1503 bp from the TSS, these sites were measured as one site. As early as 1 h after E_2_ treatment, a significant enrichment for each of the six ERE sites was observed (Fig. 5B). After 3 and 6 h of E_2_ stimulation, all of the sites showed a significant decrease in ERα occupancy as compared with 1 h of exposure to E_2_. However, after 24 h of exposure to E_2_, all of the ERE sites appeared to be once again occupied by ERα. Interestingly, the ERE located at 1004 bp from the TSS appears to be bound to ERα at all of the times tested and showed the highest level of occupancy. This pattern of ERα occupancy on the *H19* promoter is reminiscent of the cycling, on-and-off binding of ER to other ER target promoters in breast cancer cell lines (Shang *et al*. 2000, Reid *et al*. 2003).

### *H19* expression correlates with ERα expression in breast cancer tumors

**Figure 6:**
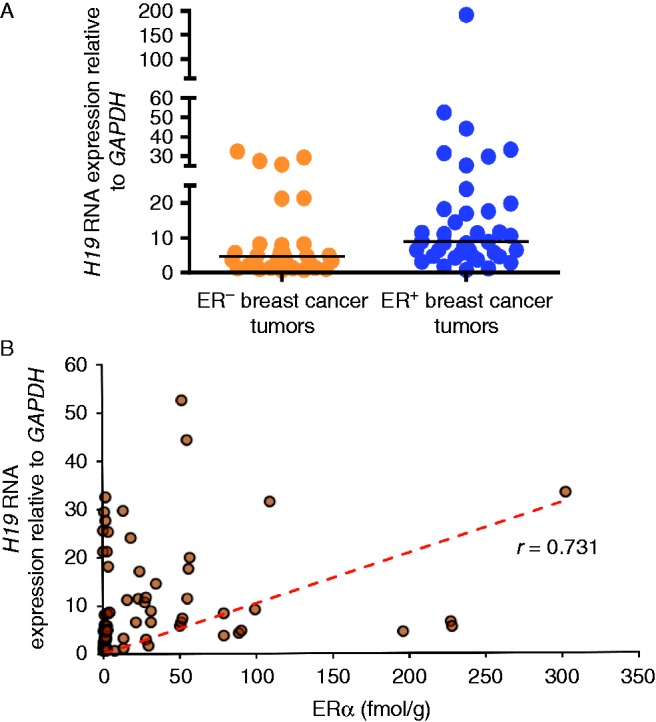
*H19* expression is associated with ER^+^ breast tumors. (A) Expression of the *H19* gene was quantified in 39 ER^+^ and 31 ER^−^ primary breast tumors using qPCR. On average, the ER^+^ breast tumors showed higher expression of *H19* (mean±s.d.=17.65±31.04) as compared with the ER^−^ tumors (8.11±9.4) as per the Mann–Whitney test (*P*=0.0030). (B) Based on a ligand binding assay, the expression of ERα was found to range from 0 to 302 fmol/mg protein. Pearson correlation revealed a positive correlation between ERα and *H19* expression in the breast tumors (*r*=0.731).

To assess the clinical relevance of our findings, we examined the expression of *H19* in 70 primary breast tumors that were obtained from postmenopausal women. Only samples that contained more than 70% tumor tissue and known ERα and PR expression levels were included. The comparative analysis of *H19* expression in these tumors using qPCR revealed a significantly higher expression of *H19* in the ER^+^ tumors (39 samples) as compared with the ER^−^ tumors (31 samples, *P*=0.0030, Fig. 6A). This observation was further validated using a larger TCGA data set that was analyzed using the UCSC Cancer Genome Browser (Supplementary Figure 8A, see section on supplementary data given at the end of this article). Next, we observed that nearly 40% of the changes in the expression of *H19* in our dataset were related to changes in ERα expression (Fig. 6B, *r*=0.73). Also, when the expression of *H19* was examined in the ERα^+^ breast tumors, a strong positive correlation was obtained (*r*=0.763, Supplementary Figure 8B), whereas no such correlation was observed when *H19* expression was examined against the ERα^low/−^ tumors (*r*=0.38, Supplementary Figure 8C). These results indicate that *H19* is expressed at a higher level in ER^+^ breast tumors as compared with ER^−^ tumors.

## Discussion

The present evidence indicates that increased estrogen signaling and increased ERα expression are significant risk factors for the development of breast cancer (Khan *et al*. 1994). Recent results have indicated that ERα^+^ cells represent a luminal-restricted progenitor cell population in healthy human breast tissue. Therefore, understanding the molecular mechanisms by which estrogen signaling regulates the proliferation and differentiation of these ERα^+^ luminal progenitors and how enhanced activation of estrogen–ERα signaling can lead to breast cancer is likely to be of value in the early detection of ER^+^ breast cancers or even in the development of preventative measures. Because of the unavailability of ERα^+^ non-malignant human breast epithelial cell lines, our current understanding of estrogen signaling at the molecular level is based on ER^+^ breast cancer cell lines. Therefore, little information is available about the targets of estrogen–ERα signaling axis in normal human breast epithelial cells. In the present report, we examined the effects of estrogen signaling on the biological functions of primary human ERα^*+*^ luminal progenitors and found that estrogen directly increased luminal progenitor cell proliferation and that blocking ERα strongly attenuated this effect of estrogen. Moreover, we also observed that co-culturing fibroblasts with luminal progenitors significantly increased progenitor numbers and that the addition of estrogen to these co-cultures had a small synergistic effect on luminal progenitor cell expansion. The present observations are in agreement with results described in previous reports that indicated that estrogen could act through paracrine effects to regulate ductal elongation in mammary glands (Mallepell *et al*. 2006, Brisken & Duss 2007, Fillmore *et al*. 2010). Our results further indicate that estrogen could act directly on ERα^*+*^ luminal progenitors and that stromal fibroblasts and the estrogen–ERα signaling axis cooperate to regulate ductal elongation during puberty. Interestingly, we found that estrogen signaling increased the expression of *H19* and ERα but not the well-identified breast cancer targets of estrogen signaling *PR* and *pS2* in matrigel cultures that had been initiated with luminal progenitors. We further showed that *H19* expression is important for estrogen-induced luminal progenitor expansion. This observation could be important to the pathogenesis of ER^+^ breast tumors, seeing as we found that *H19* is more significantly expressed in ER^+^ breast cancer tumors and that its expression is correlated with ER expression in these tumors. This finding is interesting because results of previous studies have indicated that *H19* could act as an oncogene in breast cancer cells (Berteaux *et al*. 2005). Previously, it was reported that *c-MYC*, an estrogen-regulated gene in ER^+^ breast cancer cells (Dubik & Shiu 1988), was also able to bind to and activate the transcription of the *H19* gene (Barsyte-Lovejoy *et al*. 2006), which indicates that estrogen signaling might in fact indirectly regulate the expression of *H19* through the activation of *c-MYC*. In the present report, we provide evidence that ligand-activated ERα binds to six ERE sites within the *H19* promoter and could directly activate the transcription of this gene in breast cancer cells. Although the ChIP data indicated that ERα occupies the EREs in the *H19* promoter as early as 1 h after induction with E_2_, the increase in *H19* transcript levels could only be detected after 10 h of exposure to E_2_, which indicates that the ERE half-sites may not be as transcriptionally active as full ERE sites are. Although we can potentially extrapolate to suggest that this is also the case in normal ERα^+^ luminal progenitors, because ChIP studies are only possible in ER^+^ breast cancer cells, we cannot exclude the possibility that estrogen signaling could indirectly regulate *H19* transcription and expression in healthy ER^+^ luminal progenitors. It would be interesting to investigate if *H19* and *MIR675* regulate the expression of the *GATA3* gene, which is known to play a role in breast cancer development as well as the development and maturation of luminal cells in the mammary gland. Although *H19* expression is increased in ERα^+^ luminal progenitors, the *H19* gene is expressed in undifferentiated bipotent progenitors, which indicates that *H19* may regulate the expression of a set of genes that are associated with proliferation and differentiation in both progenitor subtypes. Perhaps such a commonly expressed progenitor-associated gene set would help explain the plasticity that has been exhibited by luminal progenitors *in vitro* and *in vivo* (Visvader & Stingl 2014).

Because *H19* has been shown to have oncogenic effects in breast cancer cells, its expression has been studied in ER^+^ and triple-negative breast tumors. In these efforts, the *in situ* hybridization technique was used to examine *H19* expression in 97 breast cancer tumors (Adriaenssens *et al*. 1998). That study, however, did not yield any informative data, because *H19* expression could only be detected in three of the tumor samples, whereas all of the other samples showed only a strong stromal expression of *H19*. Most probably this was a result of the limited resolution of the *in situ* hybridization technique that was employed. In the present study, we used the sensitive and quantifiable qPCR assay to examine the expression of *H19* in ER^+^ and ER^−^ breast tumor samples that contained at least 70% tumor cells and found that *H19* expression was highly correlated with ERα expression in these tumors. Lastly, our observation that estrogen signaling in the luminal progenitors does not increase the expression of PR and *pS2*, which are classical targets of estrogen signaling in breast cancer cells, indicates that in healthy luminal progenitors, estrogen signaling could activate a different network of molecular targets to that it activates in breast cancer cells.

## Conclusion

The present results provide new insights into the molecular mechanism through which estrogen regulates human mammary gland development, and they identify *H19*, a lncRNA, as a regulator of luminal progenitor cell expansion. Our observations also lay the foundation for new studies aimed at understanding the differences in the molecular targets of estrogen signaling in healthy versus transformed primary human cell types and breast cancer cells. Based on these findings, it is possible to propose the hypothesis that some of the breast cancer-specific targets of estrogen signaling may function as early premalignant changes in the process that leads to breast cancer initiation and development. Furthermore, some of these breast-cancer-specific targets may prove useful in ascertaining the risk of developing ER^+^ breast cancer in the high-risk patient population.

## Supplementary data

This is linked to the online version of the paper at http://dx.doi.org/10.1530/ERC-15-0105.

## Author contribution statement

P Basak, L C Murphy, and A Raouf conceived of and designed the experiments. The experimental procedures were performed by P Basak, S Chatterjee, and S Weger and were analyzed by P Basak, S Chatterjee, M C Bruce, A Raouf, and L C Murphy. P Basak, L C Murphy, and A Raouf prepared the manuscript. L C Murphy and A Raouf contributed reagents, materials, and analysis tools.

## Supplementary Material

Supplementary Data
