# Supplementary material for: Estrogen regulates luminal progenitor cell differentiation through H19 gene expression
Source: Endocr Relat Cancer. 2015 Apr 13;22(4):505–17. doi: 10.1530/ERC-15-0105 (PMC4498491; doi:10.1530/ERC-15-0105)
Supplement: Supplementary Data [file supp_22_4_505__index.html]

Estrogen regulates luminal progenitor cell differentiation through H19 gene expression — Estrogen enhances luminal progenitor expansion — Supplementary Data 

# Estrogen regulates luminal progenitor cell differentiation through *H19* gene expression

## Supplementary Data

- Supplementary Figure 1 - ***ERα* is strongly expressed in the luminal progenitors** **A**. The expression of *ERα* (Green color) in the luminal or the bipotent progenitors was detected using Immunofluorescence staining. Propidium Iodide was used to mark the nucleolus (Red color). As controls, some cells were stained with mouse IgG1 (**I**). **II** – **IV**; Bipotent progenitors showed very weak staining for *ERα* (blue arrows) while the majority of the luminal progenitors (**V** – **VII**) showed strong staining for *ERα* (orange arrows). **B**. EpCAMbrightCD49low (luminal progenitors) cells were isolated from reduction mammoplasty samples and placed in colony forming cell assays. After 7 days the colonies were fixed and stained with anti Cytokeratin 18 (Ck18, green color) and Cytokeratin 14 (Ck14, red color) antibodies. DAPI staining (blue color) was used to visualize the nucleus. The colonies were visualized using a fluorescent microscope and representative picture taken at 10X magnification is shown (the white bar represents 400μm). A close up of the area boxed with red lines is also shown (image captured at 20X magnification, the white bar represents 200μm). As can be seen colonies originated from the luminal progenitors contain 100% luminal cells (i.e. all cell show Ck18 staining). C. Estrogen induces *ERα* expression in matrigel cultures initiated with luminal progenitors. Luminal progenitors were isolated as described in the materials and methods section and placed in Matrigel cultures for 7 days. The growth media was then switched to estrogen-reduced growth media for 48 hrs, after which the growth media was supplemented with estradiol (E2) or ethanol (EtOH). After 24 hrs, the gels were extracted, formalin fixed and embedded in paraffin and sections were stained with antibodies against *ERα*. FITC conjugated secondary antibody was used to detect the expression of *ERα* (Panels **II** and **IV**, Green color). DAPI was used to mark the nucleolus (blue color). Some cells were stained with mouse IgG1 to determine background staining (Panels **I** and **III**). **II** EtOH supplemented matrigels show very weak staining for *ERα* (yellow arrows), while E2 treatment of the matrigel (**IV**) shows robust staining for *ERα*. In these cells *ERα* can be detected in the nucleus as well as the cytoplasm of the cells (red arrows). D. Luminal progenitors where isolated and grown as in (C) and exposed to estrogen for 24 hrs. Subsequently, gels were dissociated and made into single cells and fixed in formalin and the expression of ER-**α** was determined via FACS. Average mean fluorescence intensity based on 3 separate experiments were obtained and presented as a bar graph. (PDF 13 KB)
- Supplementary Figure 2 - **Estrogen signaling is essential for the expansion and proliferation of luminal progenitors**. **A**. Luminal progenitors were placed in the colony forming cell (CFC) assays and either treated with 17- β estradiol (E2) or ethanol (EtOH) or ICI (a specific *ERα* blocker) at the indicated concentrations. The CFC cultures were fixed and stained and the colony numbers were quantified (N=3). As shown, in presence of E2 the colony forming capacity of luminal progenitors was enhanced (1.47 fold, compared to EtOH). Addition of ICI to the CFC cultures significantly decreased the number of colonies formed, stating at 50 nm concentration compared to the EtOH controls. **B**. *ERα* is strongly expressed in the luminal progenitors after 7 Days in matrigel. Sorted luminal progenitors were placed in matrigel cultures without irradiated fibroblasts in complete medium for 7 days when gels were dissolved and made into single cells. Cells were then fixed and permeabilized and expression of *ERα* protein was detected using flow cytometry. The histograms are representative of 3 different samples and represented as median fluorescence intensity. (\*\*\*\* p<0.0001). **C**. Sorted luminal progenitors (from two separate reduction samples) placed in matrigel cultures and treated as in (B). The single cell suspensions from these gels were dried on glass slides, fixed and stained with anti *ERα* antibody and then with a FITC conjugated secondary antibody. DAPI (Blue color) was used to stain the nucleoli. Using a fluorescent microscope, the number of *ERα*+ cells (green cells) was determined by examining 274 cells. **D**. Luminal progenitors were placed in matrigel cultures without irradiated fibroblasts in complete medium for 7 days. Subsequently gels were treated with E2 or EtOH for an additional 7 days in Phenol Red-free SF7 media. Subsequently, gels were dissolved and made into single cells, fixed and permeabilized, and stained with anti Ki67 antibody. The percentage of Ki67+ cells was determined using flow cytometry. The plots are representative of 3 independent samples. **E**. Phenotypic characterization of cells derived from luminal progenitors cultured in matrigel. Luminal progenitors were isolated from 3 different reduction samples and placed in matrigel cultures for 7 days, then supplemented with E2 or EtOH for additional 7 days. Thereafter, gels were dissociated and single-cell preparations were stained antibodies raised against CD49f, EpCAM, Cytokeratin 14 (Ck14), or Cytokeratin 8/18 (Ck18). Protein expression was obtained using flow cytometry. As shown, most of the cells retained CD49f+EpCAM+ phenotype. Majority of the cells were also positive for are Ck18 while few cells showed positive staining for both Ck14 & Ck18. The graphs are representative of three independent experiments. The average percent positive cells for each quadrant is shown in the corresponding quadrant. (\*\* p<0.008, \*\*\* p<0.0001, \*\*\*\* p<0.00001). (PDF 64 KB)
- Supplementary Figure 3 - ***H19* expression is not modulated by estrogen signaling in the matrigel cultures initiated from bipotent progenitors**. EpCAMlowCD49fbright cells (the bipotent progenitors) were isolated from reduction mammoplasty samples via Fluorescent Activated Cell Sorting (FACS) and placed in matrigel cultures under estrogen-depleted conditions. After 7 days gels were treated with estradiol (E2) or ethanol (EtOH) as control for 24 hrs and expression of *H19* (**A**.) and *ERα* (**B**.) was determined through qPCR and normalized to the *GAPDH* transcript levels. As shown, addition of E2 does not alter *H19* or *ERα* expression in these cultures. (PDF 14 KB)
- Supplementary Figure 4 - **Lenti-shH19 successfully knockdowns expression of *H19* in luminal progenitors and their progeny** Reduction mammoplasty samples were made into single-cell suspensions and infected with a pool of 3 different lentivirus expressing a short hairpin RNA against the *H19* gene or a scrambled control. The transduced luminal progenitors were isolated by fluorescent activated cell sorting and placed in colony forming cell assay as described in the Materials and Methods section. After 7 days RNA was extracted from the colonies and turned into cDNA. The *H19* transcript levels were assessed using qPCR and normalized to the *GAPDH* expression levels. As shown, transduced luminal progenitors generated progeny showed significantly decreased *H19* expression level (n=2). Because the data is based on 2 reduction samples, no error bars can be provided. (PDF 12 KB)
- Supplementary Figure 5 - **Estrogen enhances *H19* expression in ER+ breast cancer cell lines**. **A**. *H19* expression was examined in two *ERα*+ breast cancer cells (MCF-7 and T-47D). Cells were grown in estrogen-depleted growth medium for 48 hrs and subsequently treated with ethanol (EtOH) or 10 nM 17β-estradiol (E2) for 24 hrs. *H19* expression was determined using qPCR and was normalized to the *GAPDH* transcript levels. The graph depicts the average of 3 independent experiments. As expected in the presence of E2, *H19* expression was increased in the MCF-7 and the T-47D cells by 3 and 4.2 folds respectively. **B**. MCF-7 cells were cultured in estrogen-depleted growth media for 48 hrs and subsequently treated with 10 nM E2 or EtOH and RNA was extracted at the indicated time points. *H19* expression was quantified by qPCR and normalized to the *GAPDH* expression. The graph depicts mean *H19* expression from 3 independent experiments and shows that *H19* expression was significantly increased after 10 hrs of exposure to E2 and continued to rise but decreased after 48 hrs. MCF-7 (**C**.) or T-47D (**D**.) cells were cultured in estrogen-depleted growth conditions as described in A and in addition to E2 they were also treated with increasing concentrations of a specific *ERα* inhibitor, ICI, for 24 hrs. *H19* expression was determined as in A and average of 3 independent experiments is shown in the graph. As expected, blocking of *ERα* signaling attenuated estrogen-induced expression of *H19*. (PDF 32 KB)
- Supplementary Figure 6 - ***ERα* is a major contributor to estrogen-induced *H19* expression**. The *ERα* negative MDA-MB231 cells were transfected with a vector expressing full-length *ERα* and were treated with either 17-β estradiol (E2) or ethanol (EtOH) as control for 24 hrs. Expression of *ERα* (**A**.) or *H19* (**B**.) genes was determined via qPCR and was normalized with respect to the *GAPDH* transcript levels. As shown, The transfected cells show a robust expression of *ERα* and that only ligand bound *ERα* was able to significantly increase the expression of *H19*. C. MDA-MB231-ERβ cells were cultured in doxycycline (Dox) or Dox plus EtOH or Dox plus E2. ERβ (**C**.) and *H19* expression levels (**D**.) were obtained through qPCR and normalized to the *GAPDH* expression. Average of 3 experiments are shown. As can be seen, doxycycline increased ERβ expression in the transfected cells and that *H19* expression was not modulated with E2. **E**. *ERα*+ T-47D cells were grown in estrogen-reduced growth condition and treated with either EtOH or E2 (10nM), or PPT (a selective *ERα* agonist, 10nM) or DPN (a selective ERβ agonist, 10 nM) for 24 hrs. *H19* expression was ascertained using qPCR and was normalized with respect to the *GAPDH* transcript levels. Average *H19* expression based on 3 independent experiments is depicted as a bar graph. As observed, PPT significantly increased *H19* expression where DPN was ineffective (\* p<0.05, \*\* p<0.005 as compared to the EtOH treated cells). (PDF 20 KB)
- Supplementary Figure 7 - **Estrogen-induced *H19* expression requires increased transcription but not new protein synthesis**. **A**. MCF-7 cells were grown in estrogen-depleted growth conditions for 48 hrs and then treated with 17β-estradiol (E2, 10 nM) or ethanol (EtOH) E2 plus various Actinomycin D (ActD) concentrations. After 24 hrs, RNA was extracted from the cells and *H19* expression was determined using qPCR and normalized with respect to the *GAPDH* transcript levels. Average *H19* expression obtained from 3 independent experiments are shown. In the presence of ActD, E2 was unable to increase *H19* expression. **B**. Effectiveness of cycloheximide (chx) in attenuating estrogen-induced *Progesterone Receptor (PR-A and PR-B)* expression was determined. MCF-7 cells were grown in estrogen-deprived growth condition as in A. and treated with EtOH or E2, or chx plus E2 or chx alone. After 24 hrs, total protein was extracted and *PR* (both A and the B subtypes, *PR\_A* and *PR\_B*) protein expression was determined using Western Blot analysis. A representative blot is shown. The western blots (N=3) were quantified and average *PR* expression was determined using densitometry and shown as bar graph. Actin expression was used as internal loading control. As expected, treatment with chx prevents estrogen-induced *PR* (both PR\_A and PR¬\_B) expression. C. MCF-7 cells were treated as in B. and *H19* expression was determined via qPCR and normalized compared to the *GAPDH* transcript levels (N=3). As observed, treatment with chx did not attenuate estrogen-induced expression of *H19* gene. ERα+ T4-7D cells were grown in estrogen-depleted growth conditions for 48 hrs and then treated with ethanol (EtOH) or E2 (10 nM) or E2 plus Actinomycin D (ActD, 1μg/ml). After 24 hr, RNA was extracted from the cells and *H19* (**D**.) or *PR* (**E**.) transcript expression was determined using qPCR and normalized with respect to the *GAPDH* transcript levels. Average transcript expression from 3 independent experiments is presented. In the presence of ActD, E2 was unable to induce expression of *H19* or *PR*. (\* p<0.05, \*\* p<0.005, \*\*\* p<0.0005). (PDF 14 KB)
- Supplementary Figure 8 - **H19 expression correlates with *ERα* expression in the human breast tumors.** **A**. We analyzed the expression of *H19* in a gene expression TCGA dataset of invasive breast carcinoma samples (n=1215) compiled by RNAseq, using the UCSC Cancer Genome Browser. In this dataset, we found that the ER+ tumors expressed *H19* at a higher level (mean =10.96±1.37) compared to the ER- tumors (mean = 10.73±1.79) based on Mann-Whitney test (p = 0.0038). The correlation coefficient between *H19* and the *ERα* expression was determined in the ER+ (**B**) and in the *ERα*low/- (**C**) breast tumors in our sample set as described in Figure 6B (see text for details). Interestingly, while *H19* expression shows a strong positive correlation with ER+ expression (r = 0.763) no such correlation was observed with *ERα*low/- breast tumors (r = 0.38). (PDF 19 KB)
- Supplementary Table 1 - **Sequence of qPCR primers used in this study**. The table shows the primer sequences used in the ChIP experiment. The primer sequences are amplifying the half ERE sites located upstream of Transcription Start site (TSS). (PDF 38 KB)
- Supplementary Information 1 - (PDF 111 KB)
