# Supplementary figures and images for: Estrogen regulates luminal progenitor cell differentiation through H19 gene expression
Source: Endocr Relat Cancer. 2015 Apr 13;22(4):505–17. doi: 10.1530/ERC-15-0105 (PMC4498491; doi:10.1530/ERC-15-0105)

**A**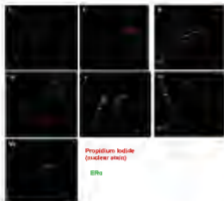**B**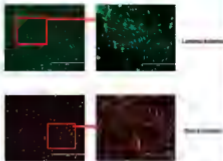**C**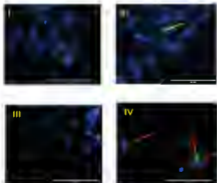**D**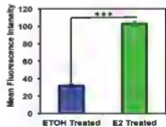

Supplement: Supplementary Data [file supp_ERC-15-0105_Supplementary_figure_1.pdf]

**A**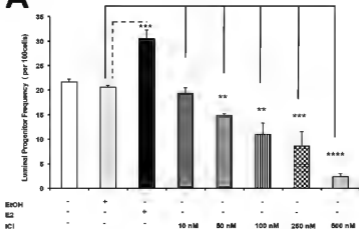**B**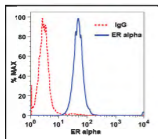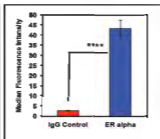**C**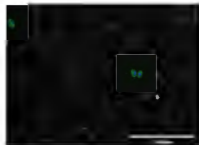**D**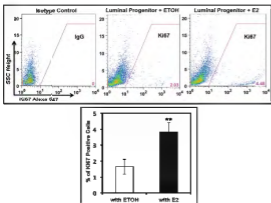**E**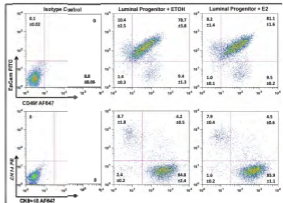

Supplement: Supplementary Data [file supp_ERC-15-0105_Supplementary_figure_2.pdf]

**A**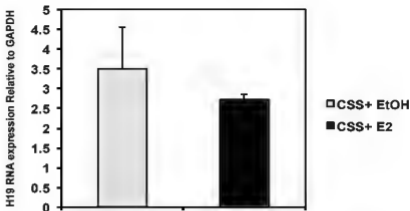**B**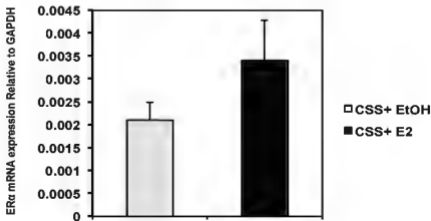

Supplement: Supplementary Data [file supp_ERC-15-0105_Supplementary_figure_3.pdf]

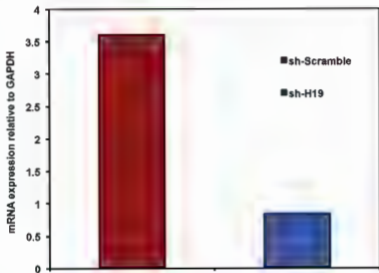

Supplement: Supplementary Data [file supp_ERC-15-0105_Supplementary_figure_4.pdf]

**A**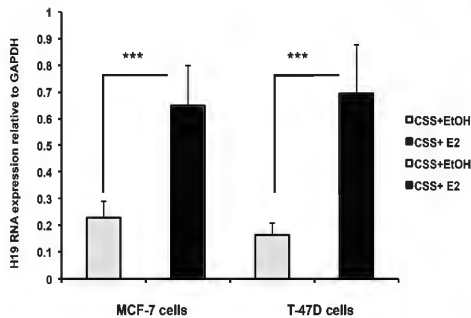**B**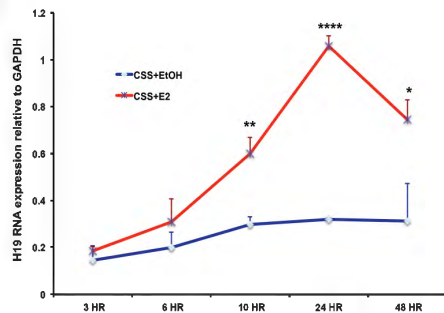**C**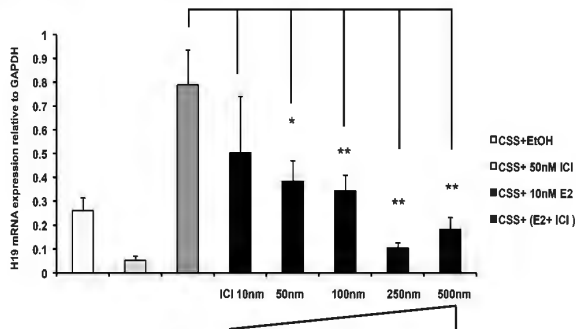**D**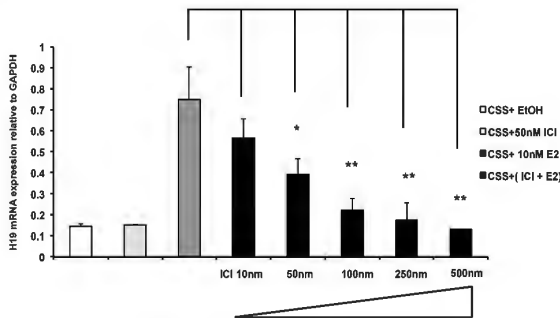

Supplement: Supplementary Data [file supp_ERC-15-0105_Supplementary_figure_5.pdf]

**A**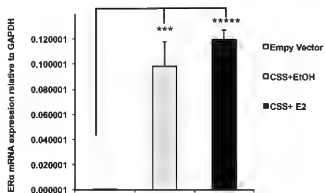**B**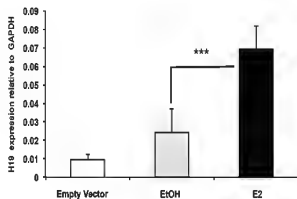**C**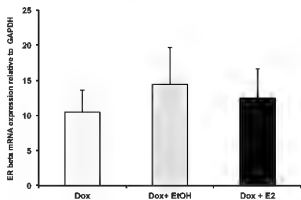**D**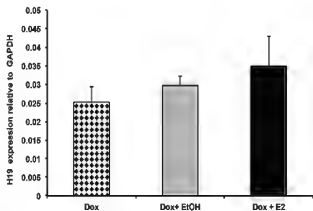**E**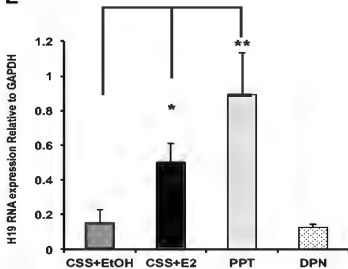

Supplement: Supplementary Data [file supp_ERC-15-0105_Supplementary_figure_6.pdf]

**A**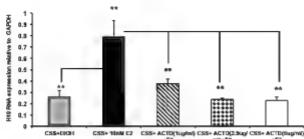**B**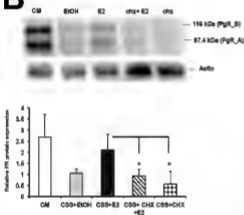**C**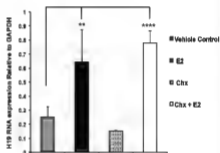**D**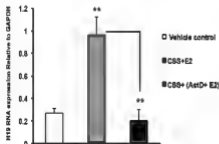**E**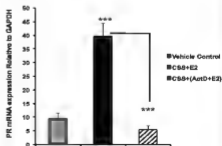

Supplement: Supplementary Data [file supp_ERC-15-0105_Supplementary_figure_7.pdf]

**A**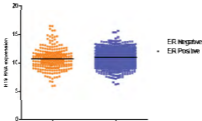**B**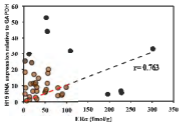**C**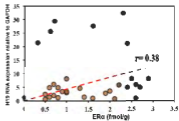

Supplement: Supplementary Data [file supp_ERC-15-0105_Supplementary_figure_8.pdf]
