# Supplementary material for: Estrogen regulates luminal progenitor cell differentiation through H19 gene expression
Source: Endocr Relat Cancer. 2015 Apr 13;22(4):505–17. doi: 10.1530/ERC-15-0105 (PMC4498491; doi:10.1530/ERC-15-0105)
Supplement: Supplementary Data [file supp_ERC-15-0105_Supplementary_table_1.pdf]

SUPPLEMENTARY TABLE 1

Supplementary Table 1. Sequence of primers utilized

Amplified Region	**FORWARD (5'-3')**	**REVERSE (5'- 3')**
+2 to +76	CGGGGGTCATCTGGGAATAG	GCATTATCCACAGCCCCGT
+32 to +136	GAGCCGCACCAGATCTTCAG	CTGGGACTCAAGTCACGCCTAC
+773 to + 902	CAGCGATGGCACAGAATCGG	TAGCAAAATGTGGGGGTCTGC
+981 to +1074	GTTCACGCACGACAAAGC	GAACAGAGGGTTTGCCGAAGG
+1172 to +1289	CTCGAGACTTGAGGTGAACCC	GGAGTCGAAACTCGCCAGTCT
+1412 to +1522*	ACAGGAAAGTGGCCGCGA	CAACTGGATGGGAATCGGC
ER non responsive region	GCCACCAGCCTGCTTTCTGT	CGTGGATGGGTCCGAGAAC
*Primers amplifying both ERE half sites		

Sequence of primers utilized. The table shows the primer sequences used in the ChIP experiment. The primer sequences are amplifying the half ERE sites located upstream of Transcription Start site (TSS).
