# Supplementary material for: Estrogen regulates luminal progenitor cell differentiation through H19 gene expression
Source: Endocr Relat Cancer. 2015 Apr 13;22(4):505–17. doi: 10.1530/ERC-15-0105 (PMC4498491; doi:10.1530/ERC-15-0105)
Supplement: Supplementary Data [file supp_ERC-15-0105_Supplementary_information_1.pdf]

SUPPLEMENTARY MATERIALS AND METHODS

Isolation of progenitors from the reduction mammoplasty samples

In our hands, the unseparated non-cultured reduction mammoplasty samples contain 22% lineage positive cells (CD35+CD45+). To enhance the frequency of progenitors, single-cell suspensions prepared from the reduction mammoplasty samples are placed in an overnight culture with 5% fetal calf serum supplemented SF7 growth media. We find that after this overnight culture the percentage of lineage positive cells falls to <1.6% of the total cells. Therefore when using pre-cultured cells to isolate luminal and the bipotent progenitors, we do not include antibodies to exclude lineage positive cells from the pre-cultured cells.

It should also be noted that the oraganoid-enriched fractions while enriched for breast epithelial cells, still contain some stromal fibroblasts that could stain positively for CD49f. We have therefore examined the expression of CD49f on fibroblasts and found that at the antibody dilution that we use (clone ID G0H3, 1:100) no significant staining for CD49f can be detected on fibroblasts.

Breast cancer cell cultures

The ER positive breast cancer cell lines MCF-7 and T-47D cells were obtained from the American Type Tissue Culture (www.ATCC.org). The MCF7 cells were maintained in Dulbecco's Modified Eagle Medium (DMEM) supplemented with 5% fetal bovine serum (FBS) and the T-47D cells were maintained in Roswell Park Memorial Institute (RPMI)-1640 media (Sigma) supplemented with bovine Insulin (Sigma) and 10% FBS. The ER negative MDA-MB231-ERβ cells were generated and maintained as previously described [unpublished data and Murphy et al. 2005).

Estrogen signaling in breast cancer cells

MCF7 cells were cultured in in PRF-DMEM media supplemented with 5% charcoal-stripped serum (v/v) 2× charcoal/dextran-treated FBS (estrogen-depleted growth media). After 48 hours, cells were treated with E2 (10 nM) or ICI (at the indicated doses) or ICI plus E2 or Actinomycin D (ActD) or cycloheximide (chx, 50 μg/ml both from Sigma Aldrich) or 4,4′,4″-(4-propyl-[1H]-pyrazole-1,3,5-triyl) trisphenol (PPT) or 2,3-bis(4-hydroxyphenyl)-propionitrile (DPN, both from Tocris Bioscience) or EtOH. After 24 hrs RNA or protein was extracted.

Immunofluorescent staining

Luminal or the bipotent progenitors were directly placed on glass slides and fixed in Acetone: Methanol (1:1) as described (Raouf et al. 2008), and stained with IgG or anti $ER\alpha$ antibody and detected by a FITC conjugated secondary antibody. PI staining was used to visualize the nucleolus. Colonies from the CFC assays were fixed with Acetone: Methanol and blocked with Dual Endogenous Enzyme Block (Dako) and were stained with antibody raised against a luminal cell marker Cytokeratin 18 (Abcam, at 1:400 dilution), and Cytokeratin 14 (Abcam, at 1:400 dilution), a myoepithelial cell marker. The expression of each protein was detected using a FITC or Cy3 conjugated secondary antibody (at 1:500 dilution). DAPI staining was used to distinguish the nucleolus. The glass slides or the plates were observed using a fluorescent microscope.

Luminal progenitors were isolated as described before and were placed in Matrigel cultures for 7 days. The cultures were then switched to estrogen-reduced growth media for 48 hrs, after which the matrigel cultures were supplemented with estradiol (E2) or ethanol (EtOH). After 24 hrs, the gels were extracted and formalin fixed and embedded in paraffin. Sections were prepared from the paraffin then blocked, deparafinized and stained with antibodies against *ERα*. FITC conjugated secondary antibody was used to detect the expression of *ERα*. DAPI was used to mark the nucleolus. For some experiments, sorted luminal progenitors from human breast reduction

samples were placed in matrigel cultures in complete medium for 7 days. After 7 days the gels were dissolved and cells were made into single cells. The cells were then fixed and permeabilized using BD Perm and Fix Kit (using manufacture's protocol). Thereafter, the cells were stained with anti *ERα* antibody (6F11, Abcam) and analyzed using a fluorescent Microscope. DAPI was used to stain the nucleoli.

RT-PCR and quantitative PCR

RNA from each sample was made into cDNA (Thermoscientific) and was used as template in Real-time Polymerase Chain Reaction (RT-PCR). For this purpose, human-specific primers were used (sequences available upon request) to detect the expression levels of *ERα*, *ERβ*, *PR*, *pS2*, *H19*, and the *GAPDH* genes in triplicates. All primers were designed to target exon-exon junctions. The relative transcript expression levels were calculated using the standard ΔCT method where normalized to the *GAPDH* expression.

Intracellular Flowcytometry

Sorted luminal progenitors from human breast reduction samples were placed in matrigel cultures in complete medium for 7 days. After 7 days the gels were dissolved and cells were made into single cells. The cells were then fixed and permeabilized using BD Perm and Fix Kit (BD Biosciences, using the manufacture's protocol). Thereafter, cells were stained with anti *ERα* antibody (6F11, Abcam) and analyzed using a bench top Flowcytometer. For some experiments, gels were treated with E2 or EtOH for an additional 7 days, when gels were dissolved single cells were obtained, fixed and permeabilized. Cells were stained with antibodies against Ki67 (Abcam), CD49f-Alexa647, EpCAM or Cytokeratin 14 (Abcam, detected with PE conjugated secondary antibody) or Cytokeratin 8/18 (Abcam, detected Alexa-647 conjugated secondary antibody) and

their expression was examined using a bench top Flowcytometer. The data was analyzed using the FlowJo software.

Western Blot Analysis

Protein lysates were prepared as described (Chatterjee et al. 2014) and 50 μg of protein was ran on a 10% SDS-PAGE gel and transferred to a membrane. Each membrane was blocked and probed with antibodies against Progesterone Receptor (*PR*) (Vector Lab, at 1:1,000) or *β-Actin* (Sigma, 1:10,000). The protein bands were detected using a Horseradish peroxidase (HRP)-conjugated secondary antibody (at 1:10,000) and visualized using HRP substrate. The protein expression was determined using *β-Actin* expression as loading control. The signal intensities were determined using the Fusion-CAPT software (Vilber Lourmat).

SUPPLEMENTARY REFERENCES

Chatterjee S, Bacopulos S, Yang W et al. 2014 Loss of igfbp7 causes precocious involution in lactating mouse mammary gland. *PLoS One.* **9** e87858.

Murphy LC, Peng B, Lewis A et al. 2005 Inducible upregulation of oestrogen receptor-beta1 affects oestrogen and tamoxifen responsiveness in MCF7 human breast cancer cells. *J Mol Endocrinol* **34** 553-566.

Raouf A, Zhao Y, To K et al. 2008 Transcriptome analysis of the normal human mammary cell commitment and differentiation process. *Cell Stem Cell* **3** 109-118.
